# Patients with Marked Prostatomegaly and Clinically Significant Prostate Cancer Have Inferior Perioperative Outcomes Following Radical Prostatectomy

**DOI:** 10.3390/jcm14227993

**Published:** 2025-11-11

**Authors:** Tara N. Morgan, Bradley Q. Fox, Austin Lai, Matthew Li, Megan Zhao, Joshua K. Kim, Jingchen Chai, David Barquin, Brian Calio, Jeffrey Gahan

**Affiliations:** 1 Department of Urology, Duke University, Durham, NC 27708, USAjeffrey.gahan@duke.edu (J.G.); 2 School of Medicine, Duke University, Durham, NC 27708, USA; bradley.fox@duke.edu (B.Q.F.);; 3 Duke Cancer Institute Biostatistics Shared Resource, Duke University, Durham, NC 27708, USA

**Keywords:** prostate cancer, radical prostatectomy, benign prostatic hyperplasia, marked prostatomegaly, prostatectomy outcomes

## Abstract

**Background/Objectives**: While robot-assisted radical prostatectomy (RARP) is the standard surgical treatment for localized prostate cancer, patients with marked prostatomegaly may experience worse outcomes. The current literature lacks generalizable, multi-surgeon data examining surgical complications in this population. **Methods**: We conducted a retrospective cohort study of 2030 patients who underwent RARP at a tertiary academic referral center. Perioperative complications and oncologic outcomes were compared between patients with marked prostatomegaly (defined as a prostate volume >100 grams (g)) and those with average-sized glands (<100 g). Logistic regression was used to compare groups. **Results**: Patients with marked prostatomegaly had a lower PSA density (0.10 vs. 0.20, *p* < 0.001), but there were no significant differences in pathologic NCCN grade groups, margin status, or lymph node involvement between groups. Patients with marked prostatomegaly had 60% higher odds of experiencing perioperative complications (OR 1.60, 95% CI 1.25–2.07, *p* < 0.0003) and were over twice as likely to have an ED visit or hospital readmission following surgery (OR 2.16, 95% CI 1.79–2.61, *p* < 0.001). They were also 25% more likely to undergo non-nerve-sparing or unilateral nerve-sparing procedures (OR 1.25, 95% CI 1.11–1.42, *p* < 0.001). **Conclusions**: Marked prostatomegaly is associated with higher rates of perioperative complications following RARP, with more frequent emergency room visits and readmissions. While nerve-sparing procedures were less commonly performed, oncologic outcomes remained comparable. Further prospective, multicenter studies are warranted to validate these results, which impact preoperative counseling.

## 1. Introduction

Prostate cancer remains one of the most common malignancies among men worldwide, with a prevalence of over 10 million people [1]. For patients with localized disease, robot-assisted radical prostatectomy (RARP) has become the standard surgical treatment. Early studies supported RARP as the gold standard across a broad range of prostate sizes. These initial studies reported largely comparable oncologic and functional outcomes between patients with marked prostatomegaly and average-sized glands [2,3]. However, more recent series suggest that patients with marked prostatomegaly actually experience worse functional outcomes following RARP, including higher rates of stress urinary incontinence and erectile dysfunction [4].

Results such as these affect all men with marked prostatomegaly, but are especially of concern in men over the age of 70 who are already at heightened risk for stress urinary incontinence and erectile dysfunction postoperatively. A cohort of over 8200 patients who underwent radical prostatectomy had functional outcomes studied by patient age at surgery and found that continence rates were higher in younger patients at the 1-year mark (93% in the <65 vs. 86% ≥ 75 years). They also found that potency rates were worse in the older patients (59% in the <65 vs. 31% ≥ 75 years) [5]. These concerns lead older patients to be more commonly treated with radiation and/or deprivation therapy (ADT) [5,6]. Yet this treatment paradigm presents challenges for men with marked prostatomegaly, as radiation may exacerbate underlying lower urinary tract symptoms (LUTS), making these patients less ideal candidates for nonsurgical approaches as well. This same dilemma exists for younger patients who are simply poor surgical candidates for radical surgery due to other factors, such as obesity.

In contemporary RARP series, fewer than 5% of patients have marked prostatomegaly over 100 grams (g) [4]. Many early studies used a lower definition of enlargement (>60–80 g) [2,3] but were still often underpowered and based on small cohorts, likely due to the relative rarity of having both clinically significant prostate cancer (PCa) and marked prostatomegaly. However, in more recent work with large sample sizes, including a systematic review, RARP outcomes in prostates over 100 g had increased surgical complexity, longer operative times, and worse postoperative functional recovery [4,7,8]. Additional findings include lower nerve-sparing rates [4], greater intraoperative blood loss [8,9], and prolonged catheterization times [4,10].

The largest study to date examining prostatectomy outcomes in this unique patient population was conducted by a single, extremely high-volume surgeon, limiting the generalizability of the reported data. To address this gap, we conducted a retrospective cohort study of RARP at an academic center, including 13 surgeons and 2030 patients. Our study also aimed to add additional findings by hypothesizing that patients with marked prostatomegaly (>100 g) and PCa undergoing RARP would experience higher rates of procedure-related complications compared to those with average-sized glands, which has not been previously reported. Secondary outcomes included readmission rates, emergency department (ED) visits, non-nerve-sparing approach rates, and oncologic outcomes. Our primary analysis aims to clarify the perioperative impact of marked prostatomegaly on RARP to better inform preoperative counseling and surgical planning using a more generalizable data set than what currently exists in the literature.

## 2. Materials and Methods

### 2.1. Study Design, Inclusion and Exclusion Criteria

After receiving approval from the Duke Health Institutional Review Board (IRB Pro00116854), we conducted a retrospective chart review of patients who underwent RARP between 2014 and 2024 at a single institution. There were 13 surgeons who performed RARP over this period at our academic referral center. Patients who opted for nonsurgical treatment, had prior bladder outlet procedures, incomplete data, nonlocalized disease, or underwent a simple rather than a radical prostatectomy were systematically excluded. In total, 2030 patients were determined to be eligible.

### 2.2. Clinical Data

Demographic data was recorded. Gleason score, grade group, surgical margin status, lymph node involvement, lymphadenectomy and nerve-sparing rates, readmission and ED visits, and final pathological stage were all obtained. Perioperative data, including duration of surgery, estimated blood loss, and perioperative complications, were noted. The Clavien–Dindo classification of each complication was recorded.

### 2.3. Defining Perioperative Complications

Given the rarity of any one single complication and the statistical power required to show a significant trend, we present herein a composite outcome which we call perioperative complications. We defined perioperative complications as any one or more of the following: ureteral injury, vesicourethral anastomotic leak or stricture, blood transfusion, bowel injury, or a prolonged anesthetic time, defined as greater than or equal to 2 standard deviations above the mean, given the substantial evidence that prolonged anesthetic time is strongly associated with and is an independent risk factor for worse perioperative outcomes [11]. More granular details of each individual complication and the associated Clavien–Dindo classification are found in Supplemental Table S1.

### 2.4. Defining Groups

The patients were divided into two cohorts: those with <100 g or >100 g on final pathology. The cut-off of 100 g was chosen based on recent studies characterizing lesion size similarly for RARP [4]. The authors believe that a cut-off of >100 g is more clinically meaningful when considering RARP outcomes than the 60–80 cc used by previous papers, which is the size cut-off in the BPH guidelines [12].

### 2.5. Prostate Size Determination

Not all patients included had the same imaging preoperatively. For consistency, the prostate size was determined based on the pathologic weight of the specimen postoperatively. Prior literature confirmed that prostate volume can be accurately estimated on preoperative imaging [13]. For those with available magnetic resonance imaging (MRI), we performed a confirmatory comparison and sensitivity analysis, both of which indicated that postoperative pathologic weight and preoperative volume were strongly correlated.

### 2.6. Statistical Analysis

Baseline demographic, oncologic characteristics, and perioperative characteristics were summarized using counts and percentages for categorical variables and medians with interquartile ranges (IQRs) for continuous variables. Participants were stratified by prostate size as above. Comparisons between groups were performed using Pearson’s Chi-squared test or Fisher’s exact test for categorical variables, and Wilcoxon rank-sum tests for continuous variables. These results are presented in Table 1, Table 2 and Table 3. The associations between perioperative complications, visits/readmissions, nerve-sparing status, and lymph node dissection (LND) status with prostate size were assessed using logistic regression. Given the imbalance in group sizes (Average Prostate: n = 1968; marked prostatomegaly: n = 61), inverse probability weighting (IPW) was applied to reduce potential bias using propensity scores estimated from a logistic regression model including body mass index, MRI PSA density, cancer grade group, and Charlson Comorbidity Index. Covariate balance before and after weighting was evaluated using standardized mean differences (SMDs), with values < 0.1 considered indicative of adequate balance. Weight distributions were summarized by mean, median, and range, and stabilized weights were truncated at the 1st and 99th percentiles to reduce the influence of extreme values. Weighted analyses were performed using robust (sandwich) variance estimators to obtain valid standard errors under the weighting scheme. To confirm, postoperative pathologic weight and preoperative volume were strongly correlated, and Pearson’s Chi-squared test was performed along with a sensitivity analysis to minimize potential classification bias. Effect estimates are reported as odds ratios (ORs) with corresponding 95% confidence intervals (CIs) and two-sided *p*-values. A *p*-value < 0.05 was considered statistically significant. All statistical analyses were performed using R version 4.4.1.

## 3. Results

Demographic data between groups can be found in Table 1. Of the 2030 patients treated with RARP at our tertiary referral center over the study period, 3.1% had prostates sized over 100 g. The median study period for the cohort was 59 months (30.1, 86.2). Patients with marked prostatomegaly were older (68 vs. 63 years, *p* < 0.001), had a slightly higher BMI (31 vs. 28, *p* = 0.002), and had higher preoperative PSA (7.8 vs. 6.3, *p* = 0.001) and median prostate volumes (44 vs. 118, *p* < 0.001). In line with being an academic medical center, there were 13 surgeons who contributed to this data set. Two surgeons contributed to 30% of cases each; one surgeon contributed 10%, and the remainder contributed ≤9% each.

To account for baseline differences between prostate size groups, inverse probability weighting (IPW) was used. After weighting, all covariates achieved SMDs < 0.1, indicating satisfactory balance between groups. After truncation at the 1st and 99th percentiles, the stabilized weights ranged from 0.86 to 1.38, with a mean of 1.00 and a median of 0.99, confirming that the weights were well-distributed around 1.0 and suggesting good model stability and adequate covariate overlap between groups.

Postoperative pathologic weight and preoperative volume on imaging were strongly correlated (Pearson’s r = 0.83, *p* < 0.01), indicating that postoperative pathologic weight served as a reliable surrogate measure of preoperative gland size. To minimize potential classification bias, a sensitivity analysis based on preoperative imaging volume produced similar results in both direction and magnitude (OR: 1.65). Patients with marked prostatomegaly had a lower preoperative prostate specific antigen density (PSAD) score (0.15 vs. 0.06, *p* < 0.001), but similar pathologic NCCN cancer grade groups, margin status, and rates of lymph node involvement to average gland sizes were observed, as shown in Table 2. There was no statistically significant difference in positive margin rates between groups.

Patients with marked prostatomegaly had 25% higher odds of receiving non-nerve-sparing or unilateral-nerve-sparing surgery compared to those with average-sized prostates (OR 1.25, 95% CI 1.11–1.42, *p* < 0.001). They had significantly higher estimated blood loss (EBL) and prolonged anesthetic times (*p* < 0.001).

When comparing RARP of an average-sized prostate, those with marked prostatomegaly had 1.60 times or 60% higher odds of experiencing perioperative complications (95% CI: 1.25–2.07, *p* < 0.0003). This is shown in Table 3. Perioperative complications are described here as a composite endpoint; Supplemental Table S1 outlines absolute numbers of complications in each group and their Clavien–Dindo classifications. Patients with marked prostatomegaly were 2.16 times more likely to have an ED visit or hospital readmission following surgery (OR = 2.16, 95% CI: 1.79–2.61, *p* < 0.001).

## 4. Discussion

Surgery and radiation therapy remain the two leading treatments for PCa, which is the most common non-cutaneous cancer in men [6,14]. Men with significant LUTS and concurrent PCa can benefit from a RARP, which offers a potential dual benefit (oncologic control, improvement in LUTS). Conversely, urinary functional decline after radiation therapy is well documented in patients with pre-existing LUTS [15].

However, RARP becomes more technically challenging in patients with marked prostatomegaly, who often have baseline LUTS. A recent expert single-surgeon experience of over 14,000 subjects found that patients with glands > 100 g had poorer functional outcomes following RARP [4]. Of note, this study was published by one of the highest volume prostate surgeons in the world, yet still a statistical decline in function over 100 g was observed. The current study builds on that experience by offering multi-surgeon, real-world data from 2030 RARP cases. We found those with marked prostatomegaly had higher odds of experiencing a perioperative complication and an ED visit or hospital readmission following surgery (*p* < 0.001). This adds to the existing literature by looking more critically at perioperative complications, building on previously reported outcomes.

There is no universally accepted definition of marked prostatomegaly in the context of RARP. We used a threshold of >100 g, consistent with the most robust prior study [4]. While earlier studies showed similar outcomes between average and large prostates, they often used a threshold of 80 g or less—a value rooted in BPH guidelines [9,10]—based on BPH surgery outcomes, but not specific to radical surgery. Another reason these lower thresholds may have been chosen is the relative rarity of this population, with under 5% estimated to have a gland size over 100 g [16], limiting the sample size at any one institution. However, given the high prevalence of PCa and the sheer number of RARPs performed in the United States, this would apply to thousands of men in the US annually.

Importantly, we observed no significant differences in oncologic outcomes between groups, whether that be final pathologic stage, grade group, margin status, or lymph node involvement. This aligns with prior studies suggesting that oncologic outcomes in patients with marked prostatomegaly are not inferior and, in fact, may have lower rates of positive surgical margins and biochemical recurrence [4,8,17]. One potential explanation may be that patients with marked prostatomegaly have PSA elevation, at least in part, from BPH, leading to risk overclassification. Current risk models do not account for PSAD [12]. This study found significantly lower PSAD in the marked prostatomegaly group, highlighting the need for more nuanced risk stratification in these patients.

Previously, associations with some outcomes of radical surgery in patients with marked prostatomegaly have been established. Men with >100 g glands have worse stress urinary incontinence (SUI) and erectile dysfunction (ED) rates following RARP [4,18,19,20]. This may be, to some extent, explained by the surgeons’ unwillingness to perform a nerve-sparing operation due to actual or perceived technical difficulties when the prostate is >100 g [4]. This finding is supported in our data, with 25% more patients not having a nerve-sparing operation if the prostate was significantly enlarged. We also found longer anesthetic times in the marked prostatomegaly group, with a nearly 30 min increase in anesthetic time. This speaks to the likely increased technical difficulty of this operation, which is reinforced by the increased estimated blood loss in our series and also previously reported [7,8].

Crucially, this study was the first to evaluate procedure-specific perioperative complications in this cohort. We found those with marked prostatomegaly had 1.60 times or 60% higher odds of experiencing a perioperative complication (95% CI: 1.25–2.07, *p* < 0.0003) and were 2.16 times more likely to have an ED visit or hospital readmission following surgery (OR = 2.16, 95% CI: 1.79–2.61, *p* < 0.001). These findings highlight the real-world risks posed by marked prostatomegaly, despite similar oncologic outcomes.

This creates a meaningful clinical dilemma. Patients with marked prostatomegaly and concurrent obstructive LUTS are poor candidates for radiation and many focal technologies, such as High-Intensity Focused Ultrasound (HIFU). Radiation therapy in patients with marked prostatomegaly has a well-known association with urinary retention and worsening of LUTS [21]. Efforts to mitigate this risk—such as robotic simple prostatectomy or laser enucleation prior to radiation—have shown promise [22], but require further evaluation in prospective series to determine functional and oncologic effectiveness.

Several limitations must be acknowledged. This was a retrospective, single-center study at an academic institution, which may limit generalizability. While the large cohort mitigates these confounders and the inclusion of 13 surgeons offers a more pragmatic, real-world perspective than prior single-surgeon series, the authors do recognize that variations in technique, experience, and institutional practices could influence outcomes, which is a limitation. An analysis of functional outcomes was omitted due to the lack of consistent quantitative data; while qualitative assessments were documented in nearly all cases, variability in reporting and interpretation was a concern. More, worse functional outcomes in patients with marked prostatomegaly have already been reported [4]. Only 3% of patients had prostates >100 g, resulting in a limited effective sample size for a weighted sub-analysis. The small number of events within each subgroup restricted model stability, increased the variance of estimated weights, and reduced the precision of effect estimates. To mitigate instability from extreme weights, stabilized inverse probability weighting (IPW) with truncation at the 1st and 99th percentiles was applied. With these measures taken together, we have shown a clear and significant increase in ED visits, readmissions, and perioperative complications in this patient population. These findings underscore the importance of careful and nuanced patient counseling and shared decision making when deciding how to treat patients with PCa and marked prostatomegaly.

## 5. Conclusions

Patients with marked prostatomegaly and PCa had higher odds of experiencing a perioperative complication, ED visit, or hospital readmission following RARP (*p* < 0.001) in a large, multi-surgeon experience. Although nerve-sparing procedures were less frequently performed in this group, oncologic outcomes remained comparable to those with average-sized glands. These findings underscore the need for enhanced preoperative counseling in patients with marked prostatomegaly who are considering prostatectomy as a treatment option. Prospective, multi-center studies are warranted to validate these findings and further inform shared decision making and individualized treatment strategies.

## Figures and Tables

**Table 1:** Demographics of Study Cohort.

Characteristic	Average Prostate (<100 g) n = 1968 ^1^	Marked Prostatomegaly (>100 g) n = 61 ^1^	*p*-Value ^2^
Age	63 (58, 67)	68 (64, 72)	<0.001
Race			0.4
White	1291 (65.6%)	35 (57.4%)	
African American	523 (26.6%)	22 (36.1%)	
Asian	33 (1.7%)	0 (0.0%)	
Undefined	121 (6.1%)	4 (6.6%)	
BMI	29 (26, 32)	31 (28, 35)	<0.001
Charlson Comorbidity Index			0.4
0	277 (14.1%)	7 (11.5%)	
1–2	1528 (77.6%)	46 (75.4%)	
≥3	163 (8.3%)	8 (13.1%)	
Preop PSA	6.3 (4.8, 9.5)	8.3 (6.1, 12.2)	<0.001
Preop MRI			0.040
Yes	1100 (55.9%)	26 (42.6%)	
No	868 (44.1%)	35 (57.4%)	
Preop SHIM Score	2 (0, 4)	4 (1, 8)	<0.001
Prostate Size	44 (35, 55)	118 (111, 134)	<0.001
Follow-up (months)	58.9 (30.1, 85.9)	67.0 (37.0, 93.0)	0.3

^1^ Median (Q1, Q3); n (%); ^2^ Wilcoxon rank sum test; Fisher’s exact test; Pearson’s Chi-squared test.

**Table 2:** Oncologic data comparing prostate glands <100 and >100 g.

Characteristic	Average Prostate (<100 g) N = 1968 ^1^	Marked Prostatomegaly (>100 g) N = 61 ^1^	*p*-Value ^2^
Preoperative PSAD	0.15 (0.11, 0.23)	0.06 (0.05, 0.09)	<0.001
Cancer Grade			0.2
Low (GG1)	193 (9.8%)	7 (11.5%)	
Intermediate (GG2 or GG3)	1555 (79.0%)	43 (70.5%)	
High (GG4 or GG5)	220 (11.2%)	11 (18.0%)	
Pathologic Stage			0.089
pT1	9 (0.5%)	2 (3.3%)	
pT2	1135 (57.7%)	35 (57.4%)	
pT3	823 (41.8%)	24 (39.3%)	
pT4	1 (0.1%)	0 (0.0%)	
Positive Margin Status			0.6
Positive	341 (17.3%)	8 (13.1%)	
Negative	1360 (69.1%)	47 (77.0%)	
Limited/Focal Positive	239 (12.1%)	5 (8.2%)	
Indeterminant	28 (1.4%)	1 (1.6%)	
Lymph Node Involvement			0.3
Yes	131 (6.7%)	2 (3.3%)	
No	1356 (68.9%)	39 (63.9%)	
No nodes taken	481 (24.4%)	20 (32.8%)	

^1^ Median (Q1, Q3); n (%); ^2^ Wilcoxon rank sum test; Pearson’s Chi-squared test; Fisher’s exact test.

**Table 3:** Summary of perioperative complications, readmissions, and emergency department (ED) visits.

Characteristic	Average Prostate (<100 g) N = 1968 ^1^	Marked Prostatomegaly (>100 g) N = 61 ^1^	*p*-Value ^2^
Perioperative Complications		1.60 (1.25, 2.07) ^1^	<0.001
ED Visits/Readmissions		2.16 (1.79, 2.61)	<0.001
EBL	150 (100, 250) ^3^	250 (150, 300)	<0.001
Length of Surgery (minutes)	235 (201, 271)	261 (221, 308)	<0.001

^1^ Odds Ratio (95% CI); ^2^ *p*-values from logistic regression; Wilcoxon rank sum test; ^3^ Median (Q1, Q3).

## Data Availability

The raw data supporting the conclusion of this article will be made available by the authors on request.

## Supplementary Materials

The following supporting information can be downloaded at: https://www.mdpi.com/article/10.3390/jcm14227993/s1, Table S1: Breakdown of the Composite End-Point, Peri-Operative Complications with Clavien-Dindo Classification.

## Abbreviations

The following abbreviations are used in this manuscript:

RARP	Robot-Assisted Radical Prostatectomy
LUTSs	Lower Urinary Tract Symptoms
ED	Emergency Department
PCa	Clinically Significant Prostate Cancer
PSAD	Prostate Specific Antigen Density
