# Supplementary material for: Patients with Marked Prostatomegaly and Clinically Significant Prostate Cancer Have Inferior Perioperative Outcomes Following Radical Prostatectomy
Source: J Clin Med. 2025 Nov 11;14(22):7993. doi: 10.3390/jcm14227993 (PMC12653873; doi:10.3390/jcm14227993)
Supplement: Supplementary file 1 [file jcm-14-07993-s001.zip › jcm-3911142-supplementary.pdf]

Supplemental Table 1. Breakdown of the Composite End-Point, Peri-Operative Complications with Clavien-Dindo Classification

Complication	Average Prostate (<100 g) N = 1,968[1]	Marked Prostatomegaly (>100 g) N = 61[1]	p- value[2]	Adj. p[3]	Clavien-Dindo Grade (% of each)
Ureteral injury	0.3% (5)	0% (0)	>0.9	>0.99	IIIb (100%)
Vesicourethral anastomotic leak	1.0% (19)	4.9% (3)	0.027	0.08	II (86%), IIIa (14%)
Vesicourethral anastomotic stricture	1.9% (37)	3.3% (2)	0.3	0.49	IIIa (31%), IIIb (69%)
Blood transfusion	2.1% (41)	1.6% (1)	>0.9	>0.99	II (100%)
Bowel injury	0.4% (7)	1.6% (1)	0.2	0.43	IIIb (100%)
Prolonged Anesthetic time	2.6% (52)	9.8% (6)	*0.007*	0.041	II (100%)
Any Complication (Overall)	7.4% (145)	18% (11)	*0.006*		

[1] % (n)

[2] Fisher's exact test

[3] Adjusted for multiple comparisons using Benjamini–Hochberg (FDR)
